# Communicable Diseases Prioritized for Surveillance and Epidemiological Research: Results of a Standardized Prioritization Procedure in Germany, 2011

**DOI:** 10.1371/journal.pone.0025691

**Published:** 2011-10-04

**Authors:** Yanina Balabanova, Andreas Gilsdorf, Silke Buda, Reinhard Burger, Tim Eckmanns, Barbara Gärtner, Uwe Groß, Walter Haas, Osamah Hamouda, Johannes Hübner, Thomas Jänisch, Manfred Kist, Michael H. Kramer, Thomas Ledig, Martin Mielke, Matthias Pulz, Klaus Stark, Norbert Suttorp, Uta Ulbrich, Ole Wichmann, Gérard Krause

**Affiliations:** 1 Robert Koch Institute, Berlin, Germany; 2 Institute of Microbiology and Hygiene, University of Saarland, Homburg, Germany; 3 Department of Medical Microbiology, University of Göttingen, Göttingen, Germany; 4 Division of Infectious Diseases, University Medical Center Freiburg, Freiburg, Germany; 5 Department for Infectious Diseases, Section Clinical Tropical Medicine, University of Heidelberg, Heidelberg, Germany; 6 Institute for Medical Microbiology and Hygiene, University of Freiburg, Freiburg, Germany; 7 Division for Communicable Diseases, AIDS and Prevention of Epidemics, Federal Ministry of Health, Berlin, Germany; 8 Family Practice “Dr. Thomas Ledig und Jansen Rolf”, Ditzingen, Germany; 9 Governmental Institute of Public Health of Lower Saxony, Hannover, Germany; 10 Department of Infectious Diseases and Respiratory Medicine, Charité University Medicine Berlin, Germany; 11 Dortmund Regional Health Authorities, Dortmund, Germany; St. Petersburg Pasteur Institute, Russian Federation

## Abstract

**Introduction:**

To establish strategic priorities for the German national public health institute (RKI) and guide the institute's mid-term strategic decisions, we prioritized infectious pathogens in accordance with their importance for national surveillance and epidemiological research.

**Methods:**

We used the Delphi process with internal (RKI) and external experts and a metric-consensus approach to score pathogens according to ten three-tiered criteria. Additional experts were invited to weight each criterion, leading to the calculation of a median weight by which each score was multiplied. We ranked the pathogens according to the total weighted score and divided them into four priority groups.

**Results:**

127 pathogens were scored. Eighty-six experts participated in the weighting; “Case fatality rate” was rated as the most important criterion. Twenty-six pathogens were ranked in the highest priority group; among those were pathogens with internationally recognised importance (e.g., Human Immunodeficiency Virus, *Mycobacterium tuberculosis*, Influenza virus, Hepatitis C virus, *Neisseria meningitides*), pathogens frequently causing large outbreaks (e.g., *Campylobacter* spp.), and nosocomial pathogens associated with antimicrobial resistance. Other pathogens in the highest priority group included *Helicobacter pylori*, Respiratory Syncytial Virus, Varicella zoster virus and *Hantavirus*.

**Discussion:**

While several pathogens from the highest priority group already have a high profile in national and international health policy documents, high scores for other pathogens (e.g., *Helicobacter pylori*, Respiratory syncytial virus or Hantavirus) indicate a possible under-recognised importance within the current German public health framework. A process to strengthen respective surveillance systems and research has been started. The prioritization methodology has worked well; its modular structure makes it potentially useful for other settings.

## Introduction

The large number of infectious agents with different pathogen-specific, host-specific and socio-economic characteristics makes the allocation of the limited resources available within the area of prevention and control of communicable diseases both challenging and controversial. The amount of attention, efforts and funds spent on surveillance, control and research of infectious pathogens varies greatly between pathogens, settings and over time. This distribution often appears to be ambiguous, potentially guided by senior leaders' research interests, short-term political agenda or residuals of historic situations [1]. As many pathogens are potentially harmful for humans and may present serious public health threats, it is necessary to prioritize the resources dedicated for surveillance and epidemiological research of infectious diseases. This needs to be done sensibly and rationally bearing in mind different aspects of pathogens' characteristics, their impact on societies and long-term consequences of their presence or introduction into populations.

The rational and transparent setting of priorities for investment into health research is therefore becoming an essential part of research planning [2]–[4]. Usefulness of prioritization, irrespective of its methodology, has been demonstrated by several research groups [5]–[11]. Prioritization can provide directions for future resource allocation and strategic planning at different levels (institutional, regional, national or international) and act as a platform for inter-disciplinary debate involving decision-makers, researchers, clinicians and the general public [8], [12].

Although today there are a number of published tools to guide the process of setting priorities, only a few publications openly describe the methodology in sufficient detail and transparency to allow reproducibility or adaptation in other settings [2], [3], [8], [13]. Furthermore, very little is published in terms of actual prioritization results.

The Department for Infectious Disease Epidemiology of the Robert Koch Institute (RKI), German national public health institute, is in charge of national surveillance, prevention, control strategies and epidemiological research in the field of infectious diseases. Together with external senior experts the Department initiated a prioritization process aiming to (1) develop a rational system for setting priorities in the area of infectious diseases using a metric-consensus approach, and (2) rank most common pathogens in accordance with their importance for national surveillance and epidemiological research to guide future work of the RKI.

## Methods

In the absence of established standards we designed a methodology using elements of our previous work in 2004 [14], [15] and experiences of other groups [2], [3], [10], [11], [13]. Our multi-staged prioritization process included compilation of the list of pathogens to be prioritized, development of evaluation criteria, weighting of criteria and scoring of the pathogens. The methodology was based on the core domain for good practices in setting priorities for research in health, such as legitimacy and fairness [3], [8], and represents the further development of the work from 2004.

### Delphi process participants

The core team (YB, AG, GK) contacted relevant leading national public health institutions with the request to name a representative to take part in the Delphi consensus process that aimed at assessing the feasibility of the methodology and the relevance of the suggested criteria, as well as to discuss possible improvements and pathogens' scores. Ten external senior experts (BG, UG, JH, TJ, MK, MKr, TL, MP, NS, UU) were nominated. They represented the National Committee of Infectious Disease Epidemiology, the German Society of Hygiene and Microbiology, the German Society of Infectious Disease Specialists, the German Society of Epidemiologists, the National Reference Laboratories, the German Federal Ministry of Health, the RKI Scientific Committee, the German Federal Chamber of Physicians and the German Medical Association. Ten internal experts (GK, AG, YB, SB, RB, TE, OH, KS, OW, MM) represented units and departments of the RKI working in the field of infectious diseases. The participants shared expertise in bacteriology, virology, mycology, parasitology, general infectious diseases, tropical medicine, general medicine, epidemiology, public health, veterinary health and infection control.

### Selection of pathogens

To maintain a broader approach, we decided to evaluate pathogens rather than diseases for their importance. A list of pathogens was compiled according to the following selection criteria: (a) notifiable according to the German law for the control of infectious diseases [16], (b) reportable within the European Union [17], (c) reportable to the WHO under the International Health Regulations [18], (d) agents with potential for deliberate release [19], and (e) pathogens represented in dedicated chapters in an established infectious diseases manual [20] and occurring in Germany. Some pathogens were grouped together when biologically and clinically plausible. The list of pathogens was reviewed by the RKI experts and the Delphi process participants.

### Prioritization criteria and scoring

The twelve criteria used during our previous prioritization process in 2004 [14], [15] were further modified according to the feedback received from a broad group of different experts [21]. The newly suggested criteria and their three-tiered definitions were then reviewed by internal and external experts. The initial scoring of all pathogens according to each criterion was performed by the core team and internal experts from the RKI. The data supporting the scoring decisions and references to the data sources, or experts' own explanations were recorded in a structured format. The internal scoring was followed by a modified two-round Delphi consensus process (internal round with RKI and joint round with additional external experts) where scores were discussed.

### Weighting

Independent of the scoring, we invited a panel of external experts to assign a weight to each criterion. This invitation was sent to all 16 federal public health institutions, all 18 national reference centers, 49 consulting laboratories, 9 scientific and professional societies working in the area of control, prevention and research of infectious diseases and to all 72 participants of the 2006 online survey [21]. External experts were asked to assign a value ranging from 0 to 10 to each criterion, thus reflecting the criterion's contextual importance for surveillance and epidemiological research. The value 0 reflects the lowest and 10 the highest level of importance of a criterion. More than one criterion could be assigned the same weight, similarly to techniques used in other prioritization exercises [5]. The final criterion's weight was defined by the median value of all weights assigned by the participating experts.

### Ranking of the pathogens

Each score was multiplied by the weight for the respective criterion. The sum of these weighted scores reflects the total weighted score of a pathogen. The total weighted scores were finally re-scaled to a range from 0 to 100 in order to facilitate final interpretation.

Following the experience from Canada [11], we did not focus on the exact numerical score assigned to a pathogen. The interpretation of the final weighted scores and their corresponding sequential ranks was done in priority groups reflecting four priority levels (the highest, high, medium and low priority). The cut-off limits for the groups were based on the equal ranges of 25 (0–25, 26–50, 51–75, 76–100). Distribution of the pathogens was later compared with their positions in the 2004 priority list.

## Results

### Selection of pathogens and scoring

In total 127 pathogens were selected for prioritization. Drug-resistant strains were scored under the common pathogen group and were not assessed as a separate entity. During the Delphi rounds, changes in pathogens' scores were made based on the consensus approach.

The Delphi process also resulted in the recommendation to remove the criterion “Emerging potential” due to its ambiguous meaning (i.e. each infectious pathogen has an ability to emerge or re-emerge) and to rephrase the definitions of some criteria. The final criteria and their definitions are presented in Table 1. The detailed scores are available in the Table S1.

**Table 1:** Prioritisation criteria and definitions of the corresponding scores.

No.	Criteria	Scoring values		
		−1	0	+1
1	Incidence (including illness and symptomatic infection)	<1/100 000	1–20/100 000	>20/100 000
2	Work and school absenteeism*	This pathogen causes a negligible proportion of absenteeism due to an infectious illness	This pathogen causes a small to moderate proportion of absenteeism due to an infectious illness	This pathogen causes a large proportion of absenteeism due to an infectious illness
3	Health care utilization (primary care and hospitalisation)*	This pathogen causes a negligible proportion of health care utilization due to an infectious illness	This pathogen causes a small to moderate proportion of health care utilization due to an infectious illness	This pathogen causes a large proportion of health care utilization due to an infectious illness
4	Chronicity of illness or sequelae*	This pathogen causes a negligible amount of chronicity or persistent sequelae (estimate prevalence of those being <0.1/100 000 population)	This pathogen causes a small to moderate amount of chronicity or persistent sequelae (estimated prevalence of those being 0.1–1.0/100 000 population)	This pathogen causes a large amount of chronicity or persistent sequelae (estimated prevalence of those being >1.0/100 000 population)
5	Case fatality rate**	<0.01%	0.01–1%	>1%
6	Proportion of events requiring public health actions (see Note 2 for explanation)**	A small proportion of the estimated total number of events or exceptional events require public health actions (<25%)	A moderate to large proportion of the estimated total number of events require public health actions (25–75%)	Almost all of the estimated total number of events require public health actions (>75%)
7	Trend**	Diminishing incidence rates	Stable incidence rates	Increasing incidence rates
8	Public attention (including political agenda and public perception)*	Risk perception of this pathogen by general public is low and it is not high on political agenda	Risk perception of this pathogen by general public is moderate and informal political expectations/agenda is present	This pathogen implies international duties or its risk perception by general public is high or it is explicitly high on political agenda
9	Prevention possibilities and needs (including vaccines)**	Preventive potential seems low or the disease does not require prevention or effective prevention strategies are well-established; no need for significant strategy modification	Measures for prevention are established but there is need to improve their effectiveness	Need for prevention is established but currently no effective preventive measures are available
10	Treatment possibilities and needs (including AMR)**	Medical treatment is rarely necessary or effective regimens are well-established; no need for significant modifications	Medical treatment regimens are established but there is need to improve their effectiveness	Need for medical treatment is established but currently no effective treatment is available or AMR limits treatment options

AMR = antimicrobial resistance.

Note 1. All criteria apply to the geographical settings where the prioritization is conducted; the time-frame applicable to the requested epidemiological data should be defined prior to the process initiation and depend on a frequency with which pathogens are planned to be re-scored. The RKI conducted re-scoring relevant for Germany using a time-frame of 5 years. Indicated numerical thresholds apply to a country where the prioritization process is conducted; when the prioritization is conducted in other geographical settings, different thresholds may need to be considered.

Note 2. Event is defined as the occurrence of a disease that is unusual with respect to a particular time, place or circumstances. For certain infectious diseases one case may be sufficient to constitute an event (e.g. polio virus). Public health actions are any kind of targeted actions aiming to identify the nature of the event and/or to apply control measures in response to the event occurrence.

*assessed against the total burden of infectious diseases.

**assessed for each particular pathogen in question, e.g., for the criterion “Treatment possibilities and needs” it therefore refers to availability and adequacy of treatment for each case of an illness caused by a particular pathogen and does not take into account the incidence of illnesses or the availability of preventive measures.

### Criteria weighting

All criteria were weighted by a total of 86 experts (14 RKI and 72 external experts). All Delphi discussion participants took part in the weighting.

The opinions regarding importance of individual criteria varied greatly across the participants for some criteria, while it was similar for others. For example, eleven participants considered the criterion “Public attention” to be of a low importance (scoring it “2”) and the same number considered it to be of a relatively high importance (scoring it “6”). At the same time, other criteria such as “Case fatality rate (CFR)” and “Trend” were weighted in a similar way by the majority of the respondents. When looking at the median weights, the criterion “Case fatality rate” was considered the most important criterion (median weight of “9”) while “Trend” and “Public attention” (both with a median weight of “5”) were considered the least important ones (Table 2).

**Table 2:** Median weight of each criteria defined by experts from different professional groups (criteria are ranked according to their priority positions among all participants).

Criterion	All participants (n = 86)	Area of professional activity		
		Epidemiologists and public health specialists (n = 43)	Laboratory experts (n = 35)	Clinicians (n = 8)
Case fatality rate	9.0	9.0	9.0	8.0
Prevention possibilities and needs	8.0	8.0	8.0	8.0
Proportion of events requiring public health actions	8.0	8.0	8.0	7.5
Chronicity of illness or sequelae	8.0	7.0	8.0	8.5
Incidence	7.0	8.0	7.0	5.5
Treatment possibilities and needs (including AMR)	7.0	6.0	8.0	7.0
Health care utilization	6.0	6.0	6.0	8.0
Work and school absenteeism	6.0	5.0	7.0	8.0
Trend	5.0	5.0	5.0	5.0
Public attention	5.0	5.0	4.0	4.0

We analyzed weights assigned by the experts working in different areas of medicine: epidemiologists and public health specialists (n = 43), laboratory specialists (n = 35) and clinicians (n = 8) (Table 2). Several criteria were assessed similarly across the groups (for example, the criteria “Prevention”, median weight of “8”, or “Trend”, median weight of “5”). At the same time, the criterion “Incidence” was seen as one of the most important by epidemiologists and public health experts (median weight of “8”) and laboratory specialists (median weight of “7”) but was seen as one of the least important by clinicians (median weight of “5.5”). Almost the reverse situation was observed with the criteria “Work and school absenteeism” and “Health care utilization” that were seen to be important by clinicians but perceived as of lower importance by the two other groups.

### Pathogen ranking

Table 3 presents the allocation of the pathogens into four priority groups according to their weighted total score. The highest priority group contains 26 (20.5%) pathogens. Among those are the pathogens that already received the highest priority in our previous prioritization process, such as HIV, *Mycobacterium tuberculosis*, *Staphylococcus aureus* including methicillin resistant strains (MRSA), Influenza virus, Hepatitis C virus, *Campylobacter* spp., *Neisseria meningitidis, Legionella pneumophila*, Varicella zoster virus (VZV). It additionally contains a number of pathogens responsible for nosocomial infections (*e.g., Klebsiella* spp., *Pseudomonas* ssp., *Enterococcus* spp.) and Respiratory syncytial virus (RSV) that were not evaluated by us previously. *Helicobacter pylori* and Hantavirus now belong to this group, while in 2004 both pathogens held medium priority positions. Other pathogens that were scored highest in 2004 were now assessed to be in the medium priority group (e.g., *Parvovirus B19*).

**Table 3:** List of pathogens in groups of priority (n = 127), Germany.

Highest priority group: scores between 76 and 100 (n = 26)	High priority group: scores between 51 and 76 (n = 39)	Medium priority group: scores between 26 and 50 (n = 45)	Low priority group: scores between 0 and 25 (n = 17)
*Campylobacter* spp	*Acinetobacter*	*Bacillus anthracis*	Actinomycosis
*Chlamydia trachomatis*	Adenovirus	*Bacillus cereus*	Astrovirus
*Clostridium difficile*	Arthropod-borne viral encephalitides	*Bartonella quintana*	*Chlamydia pneumoniae*
*Escherichia coli*, shiga toxin producing (STEC/HUS)	*Aspergillus* spp.	*Bordetella pertussis*	Coxsackievirus
*Escherichia coli* (non-gastro illnesses)	*Brucella* spp	*Borrelia burgdorferi*	*Cyclospora cayetanensis*
*Enterobacter* spp.	*Corynebacterium ulcerans* and *Corynebacterium pseudotuberculosis*	*Burkholderia cepacia*	*Entamoeba histolytica*
*Enterococcus* spp. (blood)	Prions causing Creutzfeldt Jakob Diseases	*Burkholderia pseudomallei* and *mallei*	Fungi (other)*
Hantavirus	Crimean–Congo hemorrhagic fever virus	*Candida* spp.	Helminths (flukes)**
*Helicobacter pylori*	*Cryptosporidium parvum/hominis*	*Chlamydia psittaci*	Helminths (nematodes)***
Hepatitis B virus	Dengue fever virus	*Citrobacter spp.*	Helminths (tapeworms)****
Hepatitis C virus	Early summer meningoencephalitis virus and other tick-borne meningoencephalitis viruses	*Clostridium botulinum*	HHV -6 and 7 (roseolovirus)
Human immunodeficiency virus (HIV)	Ebola and Marburg virus	*Clostridium perfringens*	*Histoplasma capsulatum*
Influenza virus	Enteroviruses spp. incl. echoviruses	*Clostridium tetani*	*Klebsiella granulomatis*
*Klebsiella* spp.	Epstein-Barr virus (HHV-4)	Coronaviruses	Molluscipoxvirus
*Legionella pneumophila*	*Giardia lamblia*	*Corynebacterium diphtheriae*	*Mycobacterium leprae*
Measles virus	*Haemophilus influenzae*	*Coxiella burnetii*	*Pneumocyctis jiroveci*
*Mycobacterium tuberculosis*	Hepatitis A virus	Cryptococcosis	Unidentified agent causing Kawasaki syndrome
*Neisseria meningitidis*	Hepatitis D virus	Cytomegalovirus (HHV-5)	
*Pseudomonas* ssp.	Hepatitis E virus	*Escherichia coli*, enteropathogenic (non STEC/HUS), enterotoxigenic strains, enteroinvasive, enteroaggregative and diffuse-adeherence strains	
Respiratory syncytial virus (RSV)	Human papilloma virus (HPV)	*Francisella tularensis*	
*Salmonella* spp. (*non-typhi* and *non- paratyphi*)	Lassa fever virus	Herpes simplex virus (HSV)-1	
*Staphylococcus aureus* incl. methicillin resistant (MRSA) and *Staphylococcus aureus*, toxigenic	*Listeria monocytogenes*	Herpes simplex virus (HSV)-2	
*Staphylococcus epidermidis*/Coagulase-negative *staphylococci*	*Microspora and trichophyton*	HHV-8 (Kaposi's sarcoma associated)	
*Streptococcus pneumoniae*	Mumps virus	Human T-cell lymphotrophic virus (HTLV)	
*Streptococcus spp*. other than *Steptococcus. pneumoniae*	*Mycoplasma* spp.	*Leishmania* spp.	
Varicella zoster virus (VZV)	*Neisseria gonorrhoeae*	*Leptospira interrogans*	
	Norovirus	*Mycobacterium*, other (non-tuberculous)	
	Parainfluenza viruses	Parvovirus B 19	
	Pediculosis (head, body and pubic lice)	*Plasmodium* spp.	
	Polio virus	Rhinoviruses	
	Rabies virus	*Rickettsia prowazekii, typhi* and *Orientia tsutsugamushi*	
	Rotavirus	*Rickettsia* spp.	
	SARS coronovirus (SARS-CoV)	Rubella virus	
	*Toxoplasma gondii*	*Salmonella paratyphi* and *Salmonella typhi*	
	Variola virus	*Sarcoptes scabiei*	
	Viruses, others causing hemorrhagic fevers (Chikungunya, Rift Valley)	*Shigella* spp.	
	West Nile virus	*Stenotrophomonas (Pseudomonas) maltophilia*	
	Yellow fever virus	*Treponema pallidum*	
	*Yersinia enterocolitica* and *pseudotuberculosis*	*Trichinella spiralis*	
		*Trichomonias vaginalis*	
		*Trypanosoma brucei gambiensi* and *brucei rhodesiensi*	
		Vaccinia virus	
		*Vibrio cholera*	
		*Vibrios (non-cholera): V. parahaemolyticus, V. vulnificus* and *V. cholerae* (non O1 and O139)	
		*Yersinia pestis*	

*Fungi (other) group includes: Blastomyces, *Fonsecaea* , *Phialophora, Cladosporium*, *Fonsecaea*, *Coccidioides immitis* and *posadasii*, *Actinomyces*, *Sporothrix*, Paracoccidioides, Zygomycota.

**Helminths (flukes) group includes: *Clonorchis sinensis, Opisthorchis felineus, Opisthorhis viverrini, Fasciolopsis buski, gigantica and hepatica, Paragonimius, Schistosoma*.

***Helminths (nematodes) group includes: *Ancylostoma braziliense* and *caninum, Angiostrongylus, Ascaris lumbricoides, Capillaria philippinensis, hepatica* and *aerophila, Dranculuse meditensis, Enterobius vermicularis, Filaria (Onchocerca volvulus, Loa loa, Wuchereria bancrofti, Brugia malayi* and *Brugia timori). hookworms (Ancylostoma duodenale* and *Necator americanus), Strongyloides stercoralis, Toxocara canis* and *cati, Trichuris trichiura. Trichinella spiralis* was scored as a separate pathogen.

****Helminths (tapeworms) group includes: *Diphyllobotrium latum, Echinococcus granulosus, Echinococcus multilocularis, Hymenolepsis nana, Taenia saginata, Taenia solium*.

The high priority group contains 39 (30.7%) and the medium priority group - 45 (35.4%) pathogens. A number of pathogens that were ranked as the least important in 2004 (*Vibrio cholera, Francisella tularensis, Bacillus anthracis, Bartonella quintana*) were now assigned to the medium and high priority groups.

Out of 17 pathogens from the low priority group in 2011, 11 were newly added.

## Discussion

This prioritization approach allowed us to benefit from the cumulative knowledge of many experts. The results of our work seem convincing to us; they support our current activities as well as indicate new directions for the future work. For example, the ranking of the majority of the pathogens found in the highest priority group (e.g., HIV, *M.tuberculosis*, Influenza virus, *Neisseria meningitides, Legionella pneumophila*) is in line with strategic goals identified by a number of international agencies that focus both on resource-constrained health systems and on industrialized countries such as Germany [4], [9], [22]–[24]. The decision to evaluate a broad range of nosocomial pathogens in the current prioritization and their high ranks indicate a growing recognition of the problem of antimicrobial resistance and healthcare associated infections (HAI) and are in line with a number of new strategic international and national policies calling for enhancement of HAI surveillance activities and capacities [4], [25].

The positioning of *Helicobacter pylori*, Hantaviruses, RSV and VZV among the pathogens in the highest priority group helped us to identify the under-recognized importance of the diseases with respect to surveillance and epidemiological research and call for actions in this respect.

Indeed, although there is a large amount of clinical and laboratory research dedicated to *Helicobacter pylori* and a number of clinical guidelines are available, very little is done in terms of public health surveillance, despite the growing rates of antimicrobial resistance seriously compromising treatment [26], [27].

RSV remains the most common respiratory pathogen in infants and young children worldwide, often resulting in serious lower respiratory tract infections [28], yet a routine surveillance system for RSV based on virological testing of sentinel respiratory samples has only been initiated recently, and the burden of disease at the population level in Germany is largely unknown. The placement of RSV in the highest priority group is particularly unusual as pathogens causing diseases in limited population groups, i.e. here in young children, are often believed to be of a lower overall importance.

The incidence of human disease caused by Hantavirus fluctuates significantly over time; its incidence has peaked in endemic areas in Germany in recent years, which may have contributed to the higher ranking of this pathogen in the 2011 prioritization and the call to enhance research activities. A population-based seroprevalence survey will indeed be initiated within the RKI-funded network of reference laboratories.

VZV, a virus that causes two frequent diseases in children and adults, was ranked as a pathogen with the highest importance both in 2004 and in 2011. Country-wide sentinel surveillance has been recently implemented for VZV after the implementation of a routine childhood Varicella vaccination program in 2004 [29].

The low priority group contains both pathogens with very low incidence in Germany (e.g., *Mycobacterium leprae* or helminths) and those much more common (e.g., Roseolovirus or *Chlamydia pneumoniae*), supporting our approach that the importance of a pathogen is defined by multiple factors rather than by its incidence or prevalence alone. Complex surveillance systems exist for some pathogens that were assigned to the medium priority group, for example, *Francisella tularensis* or *Yersinia pestis*. Although the amount of research that should be dedicated to those medium- and low-priority pathogens must be revised, undoubtedly the need to investigate outbreaks associated with these pathogens, to maintain diagnostic capacity and to continue efficient surveillance to pick up increasing trends of these rare illnesses, remains.

Similarly to other research groups that initiated processes for setting priorities [2], [8], [13], we found the compilation of the data necessary for the scoring process to be challenging due to a limited availability of reliable and published information. Participants did not find it easy to maintain sufficiently broad judgements despite the highly specialized expertise and focused research interest of some experts. Some degree of subjectivity can therefore never be avoided. Another limitation of our study was the difficulty to appropriately account for the decreasing trends of vaccine-preventable diseases that followed successful implementation of effective prevention programmes. Therefore, relatively low scoring of the respective pathogens for some of the criteria should not question the need to maintain adequate surveillance capacity for these illnesses.

In the applied methodology, we aimed at following the main principles of good practice in setting priorities in health research [3] and to reach maximum levels of objectivity, transparency, and reproducibility by integrating the following components: 1) a broad but systematic and reproducible selection of pathogens, rather than respective diseases to be included in the prioritization, 2) an explicit definition of each of the ten criteria using a three-tiered grading approach, 3) a comprehensive individual pathogen- specific scoring according to the best available evidence reviewed by a multidisciplinary expert group, and 4) a separate weighting of the criteria based on the involvement of a broad range of internal and external experts. Although this approach required intensive preparation, we believe it assured a high level of objectivity and transparency as demanded by Nuyens *et al* [7].

Our pathogen-specific approach allowed us to conduct prioritization not influenced by programmatic views and to compare pathogens' ranking within a group of diseases as well as across the groups (e.g., a pathogen being targeted by an antimicrobial resistance programme as well as by a zoonoses programme). This approach helped us uncover some differences in the importance of pathogens belonging to the same group, e.g., *Neisseria gonorrhoeae* and *Trichomonias vaginalis*.

Marked differences were observed in the weighting of the criteria between different professional groups. As the invitation to participate in the weighting was sent to various institutions and often forwarded further, it is not feasible to estimate the response rate. However, we received responses from at least one member of each contacted institution. The weighting of criteria is likely to correlate with societal values and reflect socio-economic, cultural and health system structure in a country. The fact that in Germany the criterion “Case fatality rate” was considered to be of the most importance, may reflect the high level of individualization and relative affluence of the German health care system. Comparisons to other countries are difficult since similar studies are lacking. One of the few other priority-setting initiatives with an explicit weighting procedure was conducted in Spain in which the criterion “Burden and importance of illness” was identified to be among the first three most relevant from a total of nine criteria, the other two being “Potential to change health outcomes” and “Potential to translate new knowledge into clinical or health services practices” [5]. We can conclude that weighting needs to be explicitly addressed in any prioritization exercise and it is particularly appropriate for a national public health institute in order to reflect expectations from a broad range of professionals as the respective stakeholders [30]. One way to even expand the societal perspective of the prioritization exercise could be to involve patients' representatives, similarly to how it has been done by Gooberman-Hill *et al* [31], for example.

Conclusions: The prioritization methodology presented here is based on the systematic evaluation of evidence and the involvement of a broad range of external experts. We feel that the results provide internal consistency and are plausible in the public health perspective. Our comprehensive and transparent approach makes the results defensible and shall give guidance for current needs in surveillance and epidemiological research in Germany. The list of ranked pathogens established here will serve as a reference for our mid-term strategic decisions, which will include strengthening the existing or introduction of new surveillance systems for pathogens from the high priority group (e.g., RSV, VZV or *Helicobacterpylori*) and re-consideration of the research and surveillance needs for those from the lowest priority group. It has already influenced the decision process on the need for the installation of new and continuation of existing national reference centers in Germany and the internal planning for the respective allocation of resources (GK personal communication). We plan to conduct a re-assessment of priorities within a five-year time frame based on the same methodology. The prioritization tool or its components can be applied across different areas of infectious diseases (by re-weighting prioritization criteria by different professional groups for different purposes) and in different geographical areas (by re-scoring pathogens according to their characteristics relevant for particular countries or continents). We hope that the presentation of our methodology could be helpful to other institutions that choose to prioritize their resources based on a transparent and standardized process.

## Supporting Information

Table S1 **Communicable diseases prioritized for surveillance and epidemiological research.** Results of pathogen scoring.
(XLS)
